# Utility estimations of different health states of patients with type I, II, and III spinal muscular atrophy in China: A mixed approach study with patient and proxy-reported data

**DOI:** 10.3389/fpubh.2022.1054931

**Published:** 2022-12-20

**Authors:** Jiahao Hu, Lin Zhu, Han Bao, Yuhan Liu, Huanping Xing, Qi Kang, Chunlin Jin

**Affiliations:** ^1^Shanghai Health Development Research Centre (Shanghai Medical Information Centre), Shanghai, China; ^2^Institute of Pharmaceutical Economics, Sun Yat-sen University, Guangzhou, China; ^3^Meier Advocacy & Support Centre for SMA, Beijing, China

**Keywords:** spinal muscular atrophy, vignette approach, CHU9D, EQ-5D-Y, EQ-5D-3L, utility

## Abstract

**Introduction:**

Spinal muscular atrophy (SMA) is a rare autosomal-recessive neuromuscular disease. Health state utility values (HSUV) are used in health economic evaluation regarding the desirability of health outcomes such as a certain health state or change in health states over time. There is no utility data of Chinese patients with SMA.

**Materials and methods:**

Vignettes were developed for 10 pediatric neurologists to value the utility of Chinese patients with Type I SMA. A mixed patient/proxy derived approach using EQ-5D-Y-3L, EQ-5D-3L, and CHU9D was adopted to estimate the HSUV data of patients with Type II and III SMA, including 112 patients and 301 caregivers.

**Result:**

The utility of Type I SMA patients ranged from 0.19 to 0.72 with the health state improved from “permanent ventilation” to “walking”. The utility of children patients with Type II and III SMA derived from EQ-5D-Y-3L ranged from 0.33 to 0.82 while that derived from CHU9D ranged from 0.46 to 0.75. The utility of adult patients with Type II and III SMA measured by EQ-5D-3L ranged from 0.30 to 0.83.

**Conclusion:**

The better health states the patients with SMA were in, the higher were the HSUV. The utilities derived from population with different age and disease subtypes were not statistically different when patients with SMA were in the same health states. We recommend further studies on the Chinese specific value set for EQ-5D-Y-3L and other PBMs for children to derive more robust utility data.

## Introduction

Spinal muscular atrophy (SMA) is a rare autosomal-recessive neuromuscular disease, which primarily affects children and is the leading genetic cause of infant death as well (1). It is caused by genetic deletions or mutations of the survival motor neuron (SMN) 1 gene (2), which results in progressive proximal muscle weakness, atrophy and, in the most severe types, paralysis (3). SMN2, a related gene, produces insufficient levels of stable SMN protein to compensate for the SMN1 deficiency, and the number of SMN2 copies that an individual carries is generally inversely proportional to the severity of the disease (4). The estimated worldwide incidence of SMA is 1 in 10,000 live births (1). However, the prevalence of SMA carrier is estimated to be 2.0% in China, which may be on a slow upward trend (5). SMA is divided into five clinical subtypes (0, I, II, III, IV) based on patient age at onset of disease symptoms and the highest motor function achieved (6). Type 0 is the most severe subtype with onset in utero and death before 6 months of age (7). Type I (infantile-onset) SMA is a common subtype in living patients which usually presents before 6 months of age with a life expectancy of less than 2 years. Without intervention, patients with Type I SMA are never able to sit independently (8). Type II usually onsets between 6 and 18 months of age. Patient with Type II could sit independently but never be able to walk (9). Type III normally onsets after age 18 months, with which patients may acquire independent ambulation, while some may lose the ability to walk in adulthood owing to the progressive nature of the disease (10). Type II and III SMA are known as later-onset SMA as well. Type IV SMA (adult-onset) is the rarest subtype and has the lowest morbidity and mortality, which generally occurs after the age of 20 (6).

The treatment of SMA involves multidisciplinary inputs from neurologists, respiratory specialists, gastroenterologists, geneticists, palliative care physicians, orthopedic surgeons, and physical therapists, etc., (11, 12). After 2016, the introduction of disease-modifying treatments (DMTs) brought new management options for patients with SMA. There are currently two approved DMTs for SMA in China. Nusinerisen was approved in China in 2019, is an antisense oligonucleotide that modifies SMN2 pre-messenger RNA splicing to increase functional SMN production. It needs to be intrathecally administered to patients (13). Risdiplam is a daily orally administered SMN2 splicing modifier that is distributed centrally and peripherally and increases SMN production. Risdiplam was approved in the China in 2021 for patients with SMA aged more than 2 months (14).

Though there are available DMTs in China, the high price raised concerns from the Chinese SMA community and payers, for example, the National Healthcare Security Administration (NHSA), which is responsible for reimbursement decisions in China. Health economic evaluation has been a necessary step in the annual appraisal procedure of National Reimbursed Drug List since 2019 in China.

There is currently no published health economic evaluation study on DMTs for SMA in the Chinese context. The published health economic evaluation studies from other countries used a discrete-event Markov model structure. Type I SMA was modeled separately, while Type II and III were often modeled together (15–21). Thus, outcome measurements for patients with Type I and Type II/III SMA, respectively, are needed in health economic evaluations. Quality-adjusted life-years (QALYs), which measures both the survival and health-related quality of life, is the most recommended and widely used outcome measurement in health economic evaluation (22). QALYs are calculated with health state utility values (HSUV), which could be derived with direct or indirect approaches. The direct approaches include methods like standard gambling (SG), time trade-off (TTO) and discrete choice experiment (DCE), while the indirect approach includes using generic preference-based measures (PBMs) such as EQ-5D (including EQ-5D adult and youth versions) and SF-6D. The indirect approach is recommended based on China Guidelines for Pharmacoeconomic Evaluations (23). It further recommends that EQ-5D-3L or EQ-5D-5L be used to derive utility for adults and EQ-5D-Y-3L be used to derive utility for children and adolescents (23). If there is no EQ-5D or other PBM instrument data collected from patients, mapping algorithm from disease-specific/generic HRQoL data to a generic PBM is acceptable. For example, an algorithm has been used to map EQ-5D-Y-3L utility scores from Pediatric Quality of Life Inventory (PedsQL) generic core scales (24).

There have been a few studies reporting the utility of patients with SMA published with different study designs and study locations. Chambers et al. surveyed 40 patient-caregiver pairs with Type I, II and III SMA in Australia using EQ-5D-Y-3L, while EQ-5D-3L Australian value sets were used as a proxy as EQ-5D-Y-3L value set was unavailable (25). Lloyd et al. used vignette approach by interviewing five clinical experts in SMA using EQ-5D-Y-3L and Pediatric Quality of Life (PedsQL) Neuromuscular Module to estimate the utility of health states describing different severities of Type I and II SMA (26). López-Bastida et al. reported proxy-reported utility of SMA patients derived from EQ-5D-3L (27). Malone et al. used PedsQL data collected in clinical trial and mapped it onto EQ-5D-Y-3L for patients with Type I SMA (20). Lo et al. designed a DCE to estimate the health utility for SMA treatment outcomes from a general public sample (28). Only one study reported the quality of life of Chinese children with SMA and their caregivers while PedsQL Neuromuscular Module and Family Impact Module were applied to the patients and their caregivers (29). Thus, no utility data of Chinese patients with SMA is currently available for inputs in economic evaluations.

The objective of this study was using a mixed approach of novel vignette approach and traditional questionnaire survey to estimate the utility of Chinese patients with SMA in different health states. It was the first such study with a largest number of SMA patients and would help methodological development in relevant areas in non-western countries. With validated methods and measurements, the trustworthy health utility data would help generate health economics evidence and further support NHSA's relevant reimbursement decision-makings, from which the Chinese SMA community would benefit.

## Materials and methods

### Study design

This study used a mixed approach of qualitative and quantitative methods. Patients with Type I SMA was extremely difficult to recruit due to the short life expectancy and a low prevalence. Furthermore, the patients with Type I SMA would never be able to sit independently, it was highly unlikely to recruit patients with better motor functions than sitting. Patients at such a young age do not have the literacy to complete a generic PBMs and no generic PBMs (except for HUI3, which did not have a Chinese value set) have been validated in infants younger than 2 years old. Thus, a vignette approach was adopted to measure the utility of Type I SMA patients. For Type II and III SMA patients, a mixed patient or caregiver-derived approach was adopted.

The whole process of collecting utility data was done through January to March 2022. The study design was approved by the Medical Ethics Committee of Shanghai Health Development Research Center (No. 2022004).

### Vignette approach for type I SMA

#### Defining health states for type I SMA

The health states of Type I SMA included four motor function milestones: “not sitting”, “sitting”, “standing” and “walking”, defined by Hammersmith Infant Neurological Exam-Part 2 (HINE-2), together with “permanent ventilation” (30).

#### Vignette development

A vignette is a description of the impact of a medical condition that is valued in a preference elicitation task to obtain a utility estimate. Vignettes may describe a medical condition, its treatment, and its impact on various domains of HRQoL (31). Lloyd et al. had published a study to estimate the utility of patients with Type I and Type II SMA (26). The example case study description was referred to and forward translated from English to Chinese by JH and ZL. The differences between the two translated versions were discussed by the authors to form the first Chinese case history description of “not sitting” state. The first version was further backward translated to English to ensure that the Chinese contents were accurately expressed, and the language was precise and easy to understand. The backward translated version was compared with the original English version to find the differences and further modify until all authors approved. The second version of Chinese case history description of “not sitting” state was thus formed.

Two pediatric neurologists with over 10 years' experiences in diagnosis and treatment of SMA were interviewed to validate the second Chinese case history description of “not sitting” state to ensure the content and expression was appropriate and easy to understand. Comments raised in the interviews were discussed and revised by the authors. In addition, questions about the differences of symptoms and functional limitations between health states were interviewed to develop the other case history descriptions of the resting health states. When all the Chinese health states descriptions had been developed, the two pediatric neurologists were interviewed again to test the feasibility, comprehensibility, and acceptability of the questions all the case history descriptions.

#### Valuation

Ten senior pediatric neurologists with over 10 years' experiences in diagnosis and treatment of SMA from hospitals in different cities/municipals were chosen to participate in valuation. The digital version of all the case history descriptions were sent to the participants through email. The participants were asked to read each case history description and to draw on their clinical experience to try to imagine how patients would be affected by that severity of SMA. After finishing reading all the case history descriptions, the neurologists were asked to complete a proxy assessment for all the health states using EQ-5D-Y-3L Chinese version.

### Mixed patient/proxy-derived approach for types II and III SMA

#### Defining health states for types II and III SMA

Seven discrete health states of Type II and III SMA were divided, including “not sitting”, “sitting supported”, “sitting unsupported”, “standing supported”, “standing unsupported”, “walking supported” and “walking unsupported”, with the increasing motor function. Adding “supported or unsupported” was to further explore the potential differences in utility (30). Health states related to sitting and standing were defined by the scores of Items 9 and 25 in Motor Function Measurement-32 items (MFM-32), respectively (32). The two highest levels of motor function about walking were defined as keeping with the highest independent mobility in Hammersmith Functional Motor Scale Expanded (HFMSE) (33).

#### Patient recruitment

A patient/caregiver derived approach was applied for Types II and III SMA patients. The patient recruitment was *via* snowballing sampling with the help of Meier Advocacy & Support Center for SMA, a Chinse patient advocacy group focusing on SMA, which had registered over 1,500 patients with different motor functions. Meier Advocacy & Support Group was responsible for patient recruitments through group chats, where only patients with definitive diagnosis and their caregivers could join. Participants were sent a link to the online survey (registered on www.wjx.cn, an online survey platform), which contained an electronic informed consent forms where participants had to complete a tick box indicating consent to participate prior to survey completion. The inclusion criterion was that patients aged over 4 who had been diagnosed with Types II or III SMA and were willing to participate in. The exclusion criterion was that the patients were diagnosed with other life-threatening diseases or in other conditions that were not suitable to participate in the study.

#### Questionnaire design

The questionnaire used in this phase included three parts: patients' characteristics, simplified motor function measurements and HRQoL measurements.

The first part collected information regarding patients' demographic variables (gender, age) and disease conditions (including disease subtypes, number of SMN2 copies and motor function status).

The second part adapted the abovementioned items and their answers from the MFM-32 and HFMSE into 7 corresponding statements. For example, respondents were asked the following question “which of the following description represents the best movements patients could do at present?”. If the statement “patient could sit with no upper limb support, basically maintain for more than 5 seconds and then hold hands touching for 5 sec” was chosen, that meant patients were in the health state of “sitting without support”.

The third part comprised two children friendly PBMs to collect utility data. EQ-5D-Y-3L and Child Health Utility Index-9 Dimensions (CHU9D) were used. EQ-5D-Y-3L were chosen as it was a widely used children-friendly PBM, while CHU9D was the only available pediatric-specific PBMs with a Chinese version and the affiliated value set for children and adolescents in China. EQ-5D-Y was designed for children aged from 8 to 15 years old and a proxy version for children aged 4 to 7 was also available (34). CHU9D was developed originally for children aged 7–17 while a proxy version could be used for children aged 5–7 (35). Thus, participated patients aged 5–7 were asked to complete the proxy version of EQ-5D-Y-3L and CHU9D by their main caregivers, while patients aged 8–15 could choose to use self-reported or proxy-reported version of both PBMs. The patients over 15 were asked to finish EQ-5D-3L.

### Data analysis

Based on the Chinese Pharmacoeconomics Evaluation Guideline, EQ-5D health states should be directly converted into China-specific utilities using Chinese value sets (23). Since there were no EQ-5D-Y-3L Chinese value set, an EQ-5D-3L Chinese value set were used as proxy (utility ranged from 0.1702 to 1.0000) in circumstances where EQ-5D-Y-3L were applied (36). A CHU9D China value set were used to score CHU9D data (utility ranged from −0.0855 to 1.0000) (37). Greater utility value indicated better health status in both value sets. Descriptive statistics were applied to describe the characteristics of the patients. The HSUV data of different states derived from two age subgroups were summarized as mean (standard deviations, SD) and median (interquartile range, IQR). The intraclass correlation coefficient (ICC) was calculated to assess the level of agreement between the CHU9D and the EQ-5D-Y-3L when used in patients under 16. An ICC below 0.75 implies poor to moderate agreement, whereas an ICC above 0.75 implies good agreement (38). Mann Whitney-U test was applied to explore the differences of utility of patients with similar motor functions but with different disease subtypes. Mann Whitney-U test was chosen as utility was not normally distributed and the homogeneity of variance was unknown. SPSS (IBM Corp. Released 2019. IBM SPSS Statistics for Windows, Version 26.0. Armonk, NY) was used for data analysis.

## Results

### HSUV of patients with type I SMA

All the 10 participated neurologists completed the valuation tasks. The mean utility values Type I SMA health states assessed by EQ-5D-Y-3L are shown in Table 1. The utility values of different health states range from 0.19 (the worst state “Permanent Ventilation”) to 0.72 (the best state “Walking”). The utility values increase with the improvements in motor functions.

**Table 1:** Type I SMA health states utility values.

**Health states**	**Permanent ventilation**	**Not sitting**	**Sitting**	**Standing**	**Walking**
	**Mean (SD)**				
EQ-5D-Y-3L health utility	0.19 (0.06)	0.29 (0.08)	0.39 (0.19)	0.60 (0.21)	0.72 (0.21)

### HSUV of patients with type II and III SMA

#### Characteristics of the patients

**Figure 1:**
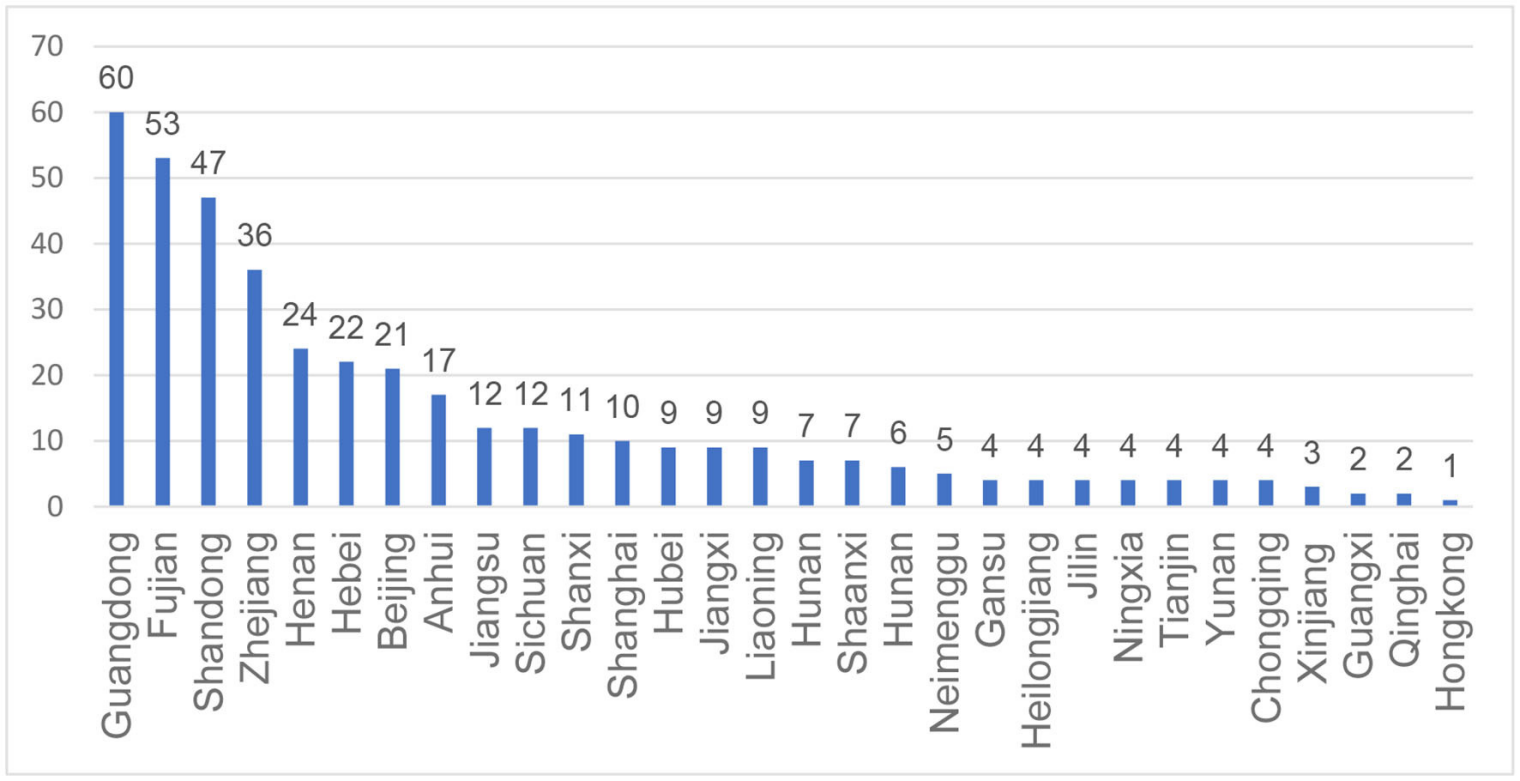
Geographical distribution of participated SMA patients.

A total of 413 patients/caregivers participated in the study. Four hundred and twelve respondents were from 29 provinces in mainland China and one was from Hongkong, no patients from Guizhou or Xizang province participated into this study. The geographical distribution of the participants was shown in Figure 1. A total of 112 patients completed the survey by themselves while the rest results were reported by the caregivers. The age of the participants ranges from 1 to 53. The mean (SD) and median (IQR) age is 13.46 (10.10) and 9 (13.00), respectively. Most patients (48.67%) had 3 SMN2 copies while 130 patients were unclear about the number of SMN2 copies. Most SMA patients (30.27%) were in the “sitting unsupported” state, followed by the “walking unsupported” state (23.49%). Only 5.57, 5.81, and 4.84% of the patients were in the “standing supported”, “standing unsupported” and “walking supported” states. The basic information about demographics and diagnosis of the patients is summarized in Table 2.

**Table 2:** Characteristics of the participated SMA patients (*N* = 413).

**Variable**	***N*** **(%)**
**Age (years old)**	
<16	288 (69.73)
≥16	125 (30.27)
**Gender**	
Male	219 (53.02)
Female	194 (46.97)
**Respondent**	
Patient	112 (27.11)
Caregiver	301 (72.89)
**Disease subtype**	
Type II	254 (61.50)
Type III	159 (38.50)
**Number of SMN2 Copies**
1	4 (0.97)
2	29 (7.02)
3	201 (48.67)
4	44 (10.65)
More than 4	5 (1.21)
Unclear	130 (31.48)
**Motor function**
Not able to sit	50 (12.10)
Sitting supported	74 (17.92)
Sitting unsupported	125 (30.27)
Standing supported	23 (5.57)
Standing unsupported	24 (5.81)
Walking supported	20 (4.84)
Walking unsupported	97 (23.49)

#### HSUV of type II and III SMA patients younger than 16

Two hundred and eighty-eight patients/caregivers used EQ-5D-Y-3L and CHU9D to derive utility value. A total of 76.39%, 71.18% and 68.75% of the the patients/caregivers reported extreme problems or unable to do in three dimensions, which are mobility, self-care, and usual activities, when completing EQ-5D-Y-3L. However, less problems were reported in the pain/discomfort and anxiety/depression dimension. When completing CHU9D, the participants similarly reported many problems/can't do in the “daily routine” and “able to join in activities” dimension, which takes 53.12% and 78.47%. respectively. The response frequencies for EQ-5D-Y-3L and CHU9D are shown in Tables 3, 4.

**Table 3:** Response frequencies (%) for the EQ-5D-Y-3L (*N* = 288).

**Dimensions**	**No problems**	**Some problems**	**Extreme problems/unable to do**
Mobility	29 (10.07)	39 (13.54)	220 (76.39)
Self-care	27 (9.38)	56 (19.44)	205 (71.18)
Usual activities	18 (6.25)	72 (25.00)	198 (68.75)
Pain/discomfort	160 (55.56)	118 (40.97)	10 (3.47)
Anxiety/depression	150 (52.08)	128 (44.44)	10 (3.47)

**Table 4:** Response frequencies (%) for the CHU9D (*N* = 288).

**Dimensions**	**Not/No. problems/any**	**A little bit/a few problems/most**	**A bit/some problem/some**	**Quite/many problems/** **a few**	**Very/can't do/NO**
Worried	101 (35.07)	67 (23.26)	33 (11.46)	44 (15.28)	43 (14.93)
Sad	143 (49.65)	3 (0.72)	5,820.14)	62 (21.53)	22 (7.64)
Pain	176 (61.11)	70 (24.31)	20 (6.94)	20 (6.94)	2 (0.69)
Tired	81 (28.13)	108 (37.50)	48 (16.67)	33 (11.46)	18 (6.25)
Annoyed	186 (64.6)	53 (18.40)	23 (7.99)	14 (4.86)	12 (4.17)
Schoolwork/ homework	139 (48.26)	42 (14.58)	29 (10.07)	17 (5.90)	61 (21.18)
Sleep	176 (61.11)	60 (20.83)	31 (10.76)	18 (6.25)	3 (1.04)
Daily routine	43 (14.93)	54 (18.75)	38 (13.19)	51 (17.70)	102 (35.42)
Able to join in activities	13 (4.51)	18 (6.25)	31 (10.76)	98 (34.03)	128 (44.44)

The average utility values of the patients under 16 with Type II and III SMA is 0.49 and 0.62 derived from EQ-5D-Y-3L and CHU-9D. With motor functions increased from “Not Sitting” to “Walking unsupported”, the average EQ-5D-Y-3L utility increased from 0.33 to 0.82, while the average CHU9D increased from 0.46 to 0.75. Notably, the CHU9D utility of “Standing supported” and “Standing unsupported” is the same, which is 0.65. ICC suggested that EQ-5D-Y and CHU9D utility indicated a moderate level of agreement. The HSUV of different health states are shown in Table 5.

**Table 5:** HSUV derived from Type II and III SMA patients under 16 using EQ-5D-Y-3L and CHU9D.

**Health state**		**EQ-5D-Y-3L utility**		**CHU9D utility**	
	***N*** **(%)**	**Mean (SD)**	**Median (IQR)**	**Mean (SD)**	**Median (IQR)**
Not sitting	32 (11.11)	0.33 (0.07)	0.32 (0.04)	0.46 (0.22)	0.45 (0.36)
Sitting supported	52 (18.05)	0.37 (0.09)	0.36 (0.06)	0.55 (0.21)	0.55 (0.33)
Sitting Unsupported	95 (32.99)	0.39 (0.10)	0.36 (0.06)	0.61 (0.20)	0.62 (0.31)
Standing supported	18 (6.25)	0.47 (0.18)	0.39 (0.25)	0.65 (0.20)	0.71 (0.32)
Standing unsupported	19 (6.60)	0.50 (0.17)	0.40 (0.26)	0.65 (0.22)	0.70 (0.28)
Walking supported	12 (4.17)	0.53 (0.18)	0.46 (0.26)	0.70 (0.21)	0.60 (0.22)
Walking unsupported	60 (20.83)	0.82 (0.16)	0.85 (0.14)	0.75 (0.18)	0.82 (0.23)
Overall^*^	288 (100)	0.49 (0.21)	0.39 (0.30)	0.62 (0.21)	0.66 (0.33)

* ICC was 0.443, *p* < 0.01.

#### HUSV of type II and III SMA patients above 16

One hundred and twenty-five patients/caregivers used EQ-5D-3L to derive utility value. Similar to patients under 16, 68.00%, 44.00%, and 54.40% of the patients above 16 reported extreme problems or unable to do in three dimensions, which are mobility, self-care, and usual activities. However, only 5 patients (4.00%) and 15 patients (12.00%) report extreme problems in the “Pain/Discomfort” and “Anxiety/Depression” dimensions, respectively. The response frequencies were shown in Table 6.

**Table 6:** Response frequencies (%) for the EQ-5D-3L (*N* = 125).

**Dimensions**	**No problems**	**Some problems**	**Extreme problems/** **unable to do**
Mobility	11 (8.80)	29 (23.20)	85 (68.00)
Self-care	38 (30.40)	32 (25.60)	55 (44.00)
Usual activities	11 (8.80)	46 (36.80)	68 (54.40)
Pain/Discomfort	53 (42.40)	67 (53.60)	5 (4.00)
Anxiety/Depression	36 (28.80)	74 (59.20)	15 (12.00)

The average utility values of the patients above 16 with Type II and III SMA is 0.56. With motor functions increased from “Not Sitting” to “Walking unsupported”, the average EQ-5D-3L utility increased from 0.30 to 0.83. The mean utility of “sitting unsupported” and “standing supported” is the same, which is 0.46. The HSUV of different health states are shown in Table 7.

**Table 7:** HSUV derived from Type II and III SMA patients above 16 using EQ-5D-3L (*N* = 125).

**Health States**		**EQ-5D-3L utility**	
	***N*** **(%)**	**Mean (SD)**	**Median (IQR)**
Not sitting	18 (14.40)	0.30 (0.71)	0.32 (0.06)
Sitting supported	22 (17.60)	0.42 (0.17)	0.35 (0.26)
Sitting unsupported	30 (24.00)	0.46 (0.16)	0.38 (0.28)
Standing supported	5 (4.00)	0.46 (0.13)	0.39 (0.24)
Standing unsupported	5 (4.00)	0.52 (0.11)	0.57 (0.21)
Walking supported	8 (8.00)	0.70 (0.11)	0.70 (0.24)
Walking unsupported	37 (29.60)	0.83 (0.14)	0.85 (0.14)
Overall	125 (100.00)	0.56 (0.24)	0.57 (0.46)

#### Comparison of utility values of patients with different subtypes and age groups

Mann-Whitney U test was used to compare the utility values of patient of similar health states with Type I, Type II and III SMA, for example, by combing “Sitting supported” and “Sitting unsupported” in Type II and III together. The same test was applied to the utility value derived from patients under and above 16 years old as well. No statistical difference was found in both analyses. The utility difference between different subtypes were shown in Table 8 and the utility difference between patients under and above 16 were shown in Table 9.

**Table 8:** The difference between utility values of Type I, Type II and III SMA patients.

	**Not sitting**	**Sitting**	**Standing**	**Walking**
Type I	0.29 (0.08)	0.39 (0.19)	0.60 (0.21)	0.72 (0.21)
Type II/III	0.33 (0.33)	0.39 (0.94)	0.49 (0.18)	0.69 (0.25)
*p* value	0.09	0.57	0.15	0.46

**Table 9:** The difference between utility values of Type II and III SMA patients under and above 16.

	**Not sitting**	**Sitting supported**	**Sitting unsupported**	**Standing supported**	**Standing unsupported**	**Walking supported**	**Walking unsupported**
EQ-5D-Y-3L	0.33 (0.72)	0.37 (0.09)	0.39 (0.10)	0.47 (0.18)	0.50 (0.17)	0.53 (0.18)	0.82 (0.16)
EQ-5D-3L	0.30 (0.71)	0.42 (0.17)	0.46 (0.16)	0.46 (0.13)	0.52 (0.11)	0.70 (0.11)	0.83 (0.14)
*p* value	0.34	0.83	0.28	0.69	0.22	0.39	0.74

## Discussion

The present study used vignette approach and mixed patient/caregiver derived approach to estimate the utility of Chinese patients with Type I, II and III SMA in different health states. As Type I, II and III SMA are the three main subtypes of SMA (39), and we recruited patients nationwide, the study could reflect the utility preference of the patients with SMA in China. Generally, we found that utility values of patients with SMA were much lower than the Chinese population norm (0.985) (40). The utility values increased with the improvements of patients' motor functions, no matter the disease subtypes or the PBMs used to measure. This finding is generally consistent with the previous studies which reported the utility of SMA patients in different health states (19, 26, 28). However, “sit/walk unaided” was the best health state and “sit/walk with assistance” was the second-best health state in Lloyd et al. (26) and Biogen's submission to National Institute for Health and Care Excellence (NICE) (19), which resulted no difference in utility for “standing with assistance” and “walking with assistance”, as well as “standing unaided” and “walking unaided” in the abovementioned two studies, respectively.

The utility values of patients in similar health states with Type I and Type II/III were slightly different but no statistical difference was found, especially the utility value of “sitting” state from Type I and Type II/III was the same. This finding may prove the validity of the vignette approach to some extent. Another possible reason was that, based on the feedbacks from pediatric neurologists participated in the valuation task, four of them claimed that they had never met a Type I SMA patient who could stand or walk, thus they brought in their experiences in treating Type II SMA patients. Furthermore, the clinicians may be familiar with several pediatric patients with SMA, which may confound their assessments of an individual case in the valuation task, which could be reason why NICE prefer utility data obtained from the person who acts as their caregiver rather than healthcare professionals (41).

We used two different PBMs for children and adolescents, which were CHU9D and EQ-5D-Y-3L to estimate the utility of adolescent patients with SMA. The EQ-5D-Y-3L utility value was on average 0.13 lower than the CHU9D utility value and the result showed CHU9D and EQ-5D-Y-3L had a moderate agreement in SMA patients. Similar results have been proven in a previous study in the population of cerebral palsy (42). We suggest that these two instruments may not be used inter-changeably to measure and value utility among children and adolescents with SMA. Though CHU9D provided another option to measure utility values in adolescents, further research would be necessary.

The utility data derived from children and adult patients with Type II and III SMA using the same value set was not statistically significant. The possible reason may be the same value set was used to derive utility. Though HTA bodies may consider adult value set acceptable for EQ-5D-Y-3L because of the lack of a validated value set, this goes against EuroQoL's recommendations (34). Japan was the only Asian country which had EQ-5D-3L and EQ-5D-Y-3L value set at the same time. The EQ-5D-Y-3L population norms for Japanese children and adolescents were lower than the EQ-5D-3L population norms for Japanese general population (43, 44), which indicated that using a proxy value set may result in the utility of adolescent patients with SMA being overestimated.

Our study has some strengths. To our best knowledge, this was the first study to estimate the utility of Chinese patients with SMA and we included patients of all the health states based on nature history of SMA. Compared with the similar studies involving patients with SMA or other rare diseases, this study had a considerably big sample size. We adopted different approaches and different reliable and validated instruments to comprehensively estimate the utility to support relevant health economic evaluations and reimbursement decisions in China as we strongly recommend that local utility data should be used to support Chinese reimbursement-decisions. Given that China is different from developed countries in socio-economic status and medical practices, it is of great significance to measure the utility of Chinse patients with SMA and further fulfill their unmet needs by valuing utility of patients with different motor functions.

Our study has several limitations worthy to notice as well. First, though vignettes in this study were developed rigorously following the good practices (31), its intrinsic methodological weakness couldn't be ignored that it may omit details that are meaningful to some patients. Furthermore, the pediatric neurologists valuated the HSUV of type I SMA patients using EQ-5D-Y proxy version. Although the applicable population age of 4–7 years is inconsistent with the age of younger patients, it is the most appropriate instrument available. Second, the patients were not evenly distributed in all the health states. The number of patients in “Standing supported”, “Standing unsupported” and “Walking supported” were significantly less than that of the other four disease states, which was due to the nature history of the disease as introduced before and the extremely high price of the DMTs. Thirdly, we adopted a mixed patient/proxy-derived utilities for patients with SMA, which may result in unintentional bias from proxy assessments. For example, parents and caregivers usually have knowledge of one child with SMA, which may skew their interpretation of a particular health state (45). Fourthly, the using of Chinese value set for EQ-5D-3L in EQ-5D-Y-3L is a consequent limitation due to the lack of Chinese specific value set for EQ-5D-Y-3L. Finally, the influencing factors of the utility values were not fully analyzed, as our main objective was to report the utility value of different health states among Chinese patients with SMA.

## Conclusion

To conclude, the better health states the patients with SMA were in, the higher were the health utilities. The utilities derived from population with different age and disease subtypes were not statistically different when patients with SMA were in the same health states. The utility generated from this study could be used in future health economic evaluations of intervention indicated for SMA. We also recommend further studies on the Chinese specific value set for EQ-5D-Y-3L and other PBMs for children to derive more robust utility data.

## Data availability statement

The original contributions of this study and important findings are included in the text, further inquiries can be directed to the corresponding authors.

## Author contributions

QK, CJ, and JH designed the survey questionnaire. HX and QK managed and coordinated the patient survey. JH and LZ developed the vignettes, was in charge of collecting survey data, and quality control. YL and HB analyzed and interpreted the survey data. JH, LZ, and YL contributed to draft the manuscript. All authors read and approved the final manuscript.
